# Chemical Profile and Phenolic Composition of Commercial Chilean Pinot Noir Wines from Clonal and Mass-Selection

**DOI:** 10.3390/plants15030359

**Published:** 2026-01-23

**Authors:** Alvaro Peña-Neira, Marco Garrido-Salinas, Karinna Estay, Oscar Seguel, Claudio Pastenes, Christián Sepulveda, Mariona Gil i Cortiella

**Affiliations:** 1 Department of Agro-Industry and Enology, Facultad de Ciencias Agronómicas, Universidad de Chile, Santa Rosa 11315, La Pintana, Santiago 8820000, Chile; karinna.estay@uchile.cl (K.E.); harvest84@hotmail.com (C.S.); 2 Department of Plant Production, Facultad de Ciencias Agronómicas, Universidad de Chile, Santa Rosa 11315, La Pintana, Santiago 8820000, Chile; marco.garrido@userena.cl (M.G.-S.); cpastene@uchile.cl (C.P.); 3 Departamento de Ingeniería y Suelos, Facultad de Ciencias Agronómicas, Universidad de Chile, Santa Rosa 11315, La Pintana, Santiago 8820000, Chile; oseguel@uchile.cl; 4 Instituto de Ciencias Aplicadas, Facultad de Ingeniería, Universidad Autónoma de Chile, Av. El Llano Subercaseaux 2801, San Miguel, Santiago 8910060, Chile; mariona.gil@urv.cat

**Keywords:** anthocyanins, tannins, proanthocyanidins, clone 115, clone 777, HPLC-DAD, phenolic acids

## Abstract

In Chile, Pinot Noir is currently cultivated on 3937 hectares, representing 7.3% of the national vineyard area dedicated to wine production. Two distinct groups of Pinot Noir plant material coexist in commercial vineyards, as officially documented by the Chilean Ministry of Agriculture: historical mass selections introduced during the 19th century (e.g., Valdivieso: Val and Concha y Toro: C&T) and certified French clonal selections (notably 115 and 777) introduced in the 1990s. Given the relevance of phenolic compounds to wine quality—particularly their role in color stability and mouthfeel—wines produced from these selections were analyzed for spectrophotometric traits and individual phenolics using high-performance liquid chromatography. Results revealed variation in parameters associated with total phenolic content and wine composition. However, no consistent differentiation between clonal and massal-derived wines was observed. Overall, the findings suggest that, under commercial winemaking conditions, the genetic origin of the planting material exerts only a limited influence on the chemical composition of Chilean Pinot Noir wines.

## 1. Introduction

Pinot Noir is one of the oldest and most esteemed grapevine cultivars worldwide, currently cultivated on more than 112,000 hectares, primarily in temperate regions [1]. Originating in the Burgundy region of France, Pinot Noir has long been associated with high-quality red wine production, and its global spread throughout the 19th and 20th centuries has made it a key variety in many New World wine regions [2]. Due to its high sensitivity to terroir, early ripening behavior, and phenotypic plasticity, Pinot Noir has become a benchmark for clonal selection and vineyard design [2].

Clonal selection is a key strategy in modern viticulture for cultivars such as Pinot Noir, which display marked phenotypic variability. It consists of propagating vines from a single mother plant exhibiting desirable traits—e.g., cluster morphology, phenology, disease tolerance, or must composition—followed by genetic stabilization and certification through multi-year field and phytosanitary evaluations. Widely adopted ENTAV-certified clones such as Pinot Noir 115 and 777 exemplify this approach due to their consistent agronomic and oenological performance. Conversely, mass selection relies on choosing cuttings from high-performing old vines within a vineyard, thereby preserving broader genetic diversity and local adaptation, albeit with less uniform outcomes [3,4,5,6,7].

In Chile, the history of Pinot Noir cultivation reflects key moments in the evolution of the national wine industry. A first wave of mass selections was introduced in the 19th century, facilitated by public and private initiatives, including the establishment of the “Quinta Normal de Agricultura”, an experimental national nursery for all manner of exotic botanical specimens, including European vines, as early as 1830, and the imports made by Silvestre Ochagavía in the 1850s [8]. These selections formed the basis of early production and led to the establishment of long-standing local materials such as “Valdivieso” and “Concha y Toro,” which are still found in some vineyards today. These early French vine imports laid the foundation for what would become Chile’s modern wine industry. They quickly supplanted traditional “Spanish varieties” such as País (syn. Listán Prieto) and Moscatel (Muscat), which had been widely cultivated during the colonial period. The adoption of these French cultivars was rapid and extensive, driven by both agronomic performance and market preferences [9]. By the end of the 19th century, the transition was well underway: in 1897, Rojas observed that “Pinot negro” (Pinot Noir) could be found in every “French vineyard” in Chile, illustrating the widespread integration of this variety into the national viticultural landscape [9].

A second wave of introductions occurred in the 1990s, in parallel with Chile’s economic liberalization and the opening of international wine markets. This period also saw the expansion of viticulture into new, cooler coastal valleys such as Casablanca. At that time, certified ENTAV clones—including 115 and 777—were introduced to respond to market demand for high-quality, terroir-expressive Pinot Noir wines [8].

A key factor that distinguishes Chile from most other wine-producing countries is its phylloxera-free status. This condition has allowed the country to maintain vineyards planted on their own roots and, more importantly, has enabled a clear and centralized registration of all introduced plant material. The Chilean Ministry of Agriculture requires that nurseries and wine producers formally declare the origin, type (massal or clonal), and identity of all vine material [10]. As a result, the genetic identity and introduction history of Pinot Noir clones such as 115 and 777 are fully documented, and the mass selections date back to known historical events [8,10,11]. This regulatory and phytosanitary framework makes Chile uniquely suited to study the chemical and phenolic differences between certified clones and historical selections, without requiring additional genetic verification.

The phenolic composition of grapes and wines, including anthocyanins, flavan-3-ols, flavonols, hydroxybenzoic acids, hydroxycinnamic acids, and stilbenes, plays a central role in determining sensory attributes such as color, body, astringency, bitterness, and aging potential [11,12]. These compounds are strongly influenced by genotype (clone or selection), environmental conditions, vineyard practices, and harvest maturity [13,14]. Several studies have shown that different clones of Pinot Noir can differ significantly in phenolic content and composition, leading to measurable differences in wine style and sensory profile [4,5,6,7,13,14].

Clonal variability plays a crucial role in determining the phenolic composition of grape berries and the resulting wines. In a five-year study conducted in Oregon, Castaglioni and Vaconcelos [5] evaluated 20 Pinot noir clones and reported a wide range in anthocyanin concentrations among them; the clone with the highest average anthocyanin level contained approximately twice the amount found in the lowest. Similarly, Burin et al. [15] investigated the phenolic composition of wines produced from two clones of Cabernet Sauvignon and found marked differences associated with clonal origin. Clone 169 was characterized by a higher presence of total polyphenols and polymerized polyphenols, whereas clone 685 consistently exhibited a predominance of anthocyanins, regardless of vineyard location. These findings, supported by multivariate analysis, suggest that phenolic profiling can serve as a reliable approach for wine classification and for distinguishing between grapevine clones.

Although the present work focuses on the chemical and phenolic composition of commercial Pinot Noir wines, its relevance extends directly to viticulture, as the comparison between certified clones and traditional mass selections provides insight into intra-varietal diversity and the genetic resources currently used in Chilean vineyards. This perspective aligns with current efforts to understand how planting material shapes grapevine performance and, ultimately, wine composition, thereby contributing to broader discussions on vineyard genetic resources and their practical implications. It is important to note that this study constitutes an observational survey of commercial wines rather than a controlled clonal experiment, which allows the assessment of plant-material effects within the realistic variability of industrial-scale production.

Despite the central role of phenolic composition in defining the style and quality of Pinot Noir wines, no published studies have compared the phenolic profiles of wines produced from these traditional Chilean mass selections with those derived from modern French clones. To address this gap, the present study characterizes the chemical and phenolic composition of commercial wines made from the “Valdivieso” and “Concha y Toro” mass selections and from clones 115 and 777, with the aim of evaluating how genetic origin and selection history influence phenolic expression under Chilean growing conditions. Within the observational nature of the dataset, this approach provides a first insight into how these distinct sources of planting material manifest in the composition of commercially produced Pinot Noir.

## 2. Materials and Methods

### 2.1. Solvents and Chemicals

Solvents and chemicals were purchased from several vendors. High-purity standards such as gallic acid, protocatechuic acid, caffeic acid, caftaric acid, *trans*-resveratrol, (+)-catechin, (−)-epicatechin, quercetin, and malvidin-3-glucoside were purchased from Sigma-Aldrich Chemical Co. (St. Louis, MO, USA) along with methylcellulose (viscosity: 1500 cP at 20 g/L). Sodium sulfate (anhydrous), vanillin, ethyl acetate, potassium metabisulfite, diethyl ether, sodium hydroxide, hydrochloric acid, sulfuric acid, and HPLC-grade solvents such as acetonitrile, acetic acid, formic acid, and methanol were acquired from Merck (Darmstadt, Germany), while polyethylene membranes with a pore size of 0.22 μm were acquired from EMD Millipore (Billerica, MA, USA), whereas Merck (Darmstadt, Germany) supplied the anhydrous sodium sulfate, vanillin, ethyl acetate, potassium metabisulfite, diethyl ether, sodium hydroxide, hydrochloric acid, sulfuric acid, and HPLC-grade solvents such as methanol, acetonitrile, acetic acid, and formic acid.

Solid-phase extraction cartridges were purchased from Waters (Milford, MA, USA), including the Sep-Pak Plus Environmental C_18_ cartridges (900 mg) and the Sep-Pak Plus Short C_18_ cartridges (400 mg). Phosphate for the buffer solution (pH 7) was obtained from Mallinckrodt Baker (Phillipsburg, NJ, USA), and nitrogen gas was supplied by Indura SA (Santiago, Chile). All chemicals and reagents used were of analytical grade or higher.

### 2.2. Red Wine Samples

Twenty-six commercial monovarietal Pinot Noir wines from the 2018 vintage, produced from different clonal and mass-selection plant materials and originating from several Chilean regions, were analyzed. The dataset comprised wines from two certified clones—115 (*n* = 5) and 777 (*n* = 6)—and two historical mass selections—Concha y Toro (C&T, *n* = 8) and Valdivieso (*n* = 7). All wines were vinified at an industrial scale in collaborating cellars located within five Chilean Protected Designations of Origin (PDO): Casablanca (*n* = 8), Limarí (*n* = 5), Central Valley (*n* = 7), Biobío (*n* = 3), and San Antonio (*n* = 3).

Grapes were harvested at technological maturity (~22–24 °Brix), with harvest dates varying by region between 15 February and 15 March. In all cases, clusters were destemmed prior to fermentation, and no whole-cluster material was used. Alcoholic fermentation was conducted in closed stainless-steel tanks under conventional on-skin vinification conditions at 23–25 °C, using commercial Saccharomyces cerevisiae (Lallemand EC-1118, Santiago, Chile) at 10–15 g/hL. Pump-over operations were applied with low mechanical intensity and limited frequency to ensure gentle cap management and avoid excessive extraction of seed-derived tannins, a key consideration in Pinot Noir vinification. Fermentative maceration proceeded until residual sugar levels dropped below 2 g/L, followed by a three-day post-fermentative maceration at 10 °C. Fermentation progress was monitored through relative density measurements.

Malolactic fermentation occurred spontaneously in all wines, and completion was verified by paper chromatography and enzymatic L-malic acid quantification (<0.1 g/L) (Megazime, Vinotec, Chile). After MLF, molecular SO_2_ was adjusted to 0.8 mg/L, and the wines were bottled and stored for approximately one month prior to analysis.

Although the collaborating wineries applied their own commercial protocols, these wines reflect standard industrial practices within each cellar; therefore, the study is explicitly observational rather than experimental.

#### Viticultural Regions and Soil Characteristics

The wines analyzed in this study originated from five Chilean viticultural zones—Limarí, Casablanca, Leyda–San Antonio, Biobío, and Austral—each with distinctive soil and climatic features [16] known to influence vine growth and berry composition, thereby contributing to the variability observed among the commercial wines included in this survey (see Supplementary Table S1 for detailed edaphic descriptors).

### 2.3. Basic Chemical Analysis and Spectrophotometric Measurement

Acidity (expressed as grams of H_2_SO_4_ per liter) and pH levels were tested using methods recommended by the International Organization of Vine and Wine (OIV) [17]. Absorbance was measured with a Hewlett Packard UV-Vis 1700 Pharmaspec spectrophotometer manufactured by Shimadzu (Kyoto, Japan). Color intensity (CI) and hue were determined following the method described by Ribèreau-Gayon et al. (2006) [12]. The total tannin content was measured according to the method proposed by Bate Smith (1981) [18], total anthocyanin content was determined using an anthocyanin assay, and total phenol content was assessed by measuring the OD 280 value [12] and applying a standard curve prepared with gallic acid.

### 2.4. Fractionation of Proanthocyanidins

Proanthocyanidins were separated according to their degree of polymerization using Sep-Pak tC18 cartridges. To begin, 7 mL of wine was evaporated to dryness at temperatures below 30 °C using a rotary evaporator. The dry residue was then reconstituted in 20 mL of phosphate buffer (67 mmol/L, pH 7). Following pH adjustment to 7 under a nitrogen atmosphere, the solution was passed through two neutral Sep-Pak tC18 cartridges, previously conditioned and arranged in series. The setup included a Sep-Pak Plus Environmental tC18 cartridge (900 mg) placed above a Sep-Pak Plus Short tC18 cartridge (400 mg). This method of fractionation is based on the procedure outlined by Sun et al. (1998) [19], with additional modifications proposed by Cáceres-Mella et al. (2014) [20].

Each collected fraction (monomeric, oligomeric, and polymeric) was then analyzed for flavan-3-ol content using a modified version of the vanillin assay, as described by Sun et al. (1998) [19]. The absorbance of each fraction was recorded at 500 nm, using methanol as the blank in place of the vanillin reagent.

### 2.5. Individual Phenolic and Anthocyanin Profiles by HPLC-DAD

Anthocyanin and low molecular phenolic compounds were analyzed using an Agilent 1100 Series HPLC system (Agilent Technologies, Santa Clara, CA, USA), which included an autosampler, quaternary pump, degasser, column oven, and a photodiode array detector (DAD). For anthocyanin separation, the mobile phase consisted of solvent A (water with 10% formic acid) and solvent B (acetonitrile). A sample injection volume of 150 μL was used. Separation was carried out on a LiChroCart C_18_ reversed-phase column (250 mm × 4.0 mm I.D., 5 μm; Merck, Darmstadt, Germany). The chromatographic method followed the protocol established by Fanzone et al. (2012) [21]. Prior to HPLC analysis, all samples were filtered through 0.22 μm pore-size membranes. Qua, ntification was conducted at 520 nm, and results were expressed as malvidin-3-glucoside equivalents.

To extract low-molecular-weight phenolic compounds, a 50 mL wine aliquot was subjected to liquid–liquid extraction using diethyl ether (3 × 20 mL) and ethyl acetate (3 × 20 mL), following the protocol described by Peña-Neira et al. (2007) [22]. The combined organic phases were dried over 2.5 g of anhydrous sodium sulfate and then concentrated to dryness under vacuum at 30 °C. The resulting residue was reconstituted in 2 mL of a methanol/water mixture (1:1, *v*/*v*) and filtered through a 0.22 μm membrane filter. Chromatographic analysis was carried out using a reverse-phase Nova-Pak C_18_ column (300 mm × 3.9 mm I.D.; 4 μm; Waters, Milford, MA, USA), thermostatted at 20 °C for HPLC-DAD analysis. Identification of low-molecular-weight phenolics was based on UV–Vis spectral characteristics (recorded from 210 to 600 nm, 2.0 nm bandwidth) and comparison of retention times with those of authentic standards. Quantification was performed using calibration curves constructed at 280 and 360 nm. For compounds lacking commercial standards, such as proanthocyanidins and stilbene glycosides, quantification was performed using calibration curves for (+)-catechin and *trans*-resveratrol, respectively.

### 2.6. Statistical Analysis

The assumptions of normal distribution of residuals and homoscedasticity of variance were tested using the Shapiro–Wilk test (rstatix R package, version 4.3.3, R Core Team, 2025, Viena, Austria) and Bartlett’s test (stats R package), respectively. The measured variables were analyzed considering, as a fixed factor, either the origin of the wine samples (clones 115 and 777; mass selections C&T and Val) or the selection method (clonal or massal) when appropriate. When assumptions were met, analyses were performed using one-way analysis of variance (ANOVA), and when they were not, the Kruskal–Wallis test was used. In cases where significant effects were detected, post hoc comparisons were conducted using Tukey’s test (parametric analysis) and Dunn’s test with Bonferroni adjustment (non-parametric). In both cases, the rstatix R package was used [23]. The significance level was set at 0.05 for all analyses.

All variables were visualized in boxplots, depicted as follows: the line inside the box represents the median, while whiskers indicate the lowest and highest values within a 1.0 interquartile range (IQR). The means, standard deviations, and ranges were considered for each cutting separately.

To explore multivariate patterns and assess the influence of selection type and geographic origin, principal component analysis (PCA) was performed using the factoextra R package [24]. All physical and chemical variables were included. The first two principal components were visualized in biplots: one showing the distribution of the wines and another displaying the variable loadings, where only the 15 highest-contributing variables were retained for clarity.

A correlation matrix of all numerical variables was also computed using the cor() function, with *p*-values obtained via the cor_pmat() function from the rstatix package. The results were visualized with the ggcorrplot() function from the ggcorrplot package [25], generating a heatmap that displays correlation coefficients alongside their statistical significance (*p* < 0.05).

## 3. Results and Discussion

This study compared the chemical and phenolic composition of commercial Pinot Noir wines produced at an industrial scale from two certified clones (115 and 777) and two traditional Chilean mass selections (Valdivieso and Concha y Toro), sourced from different wineries.

### 3.1. Basic Chemical Composition of the Wines

**Figure 1:**
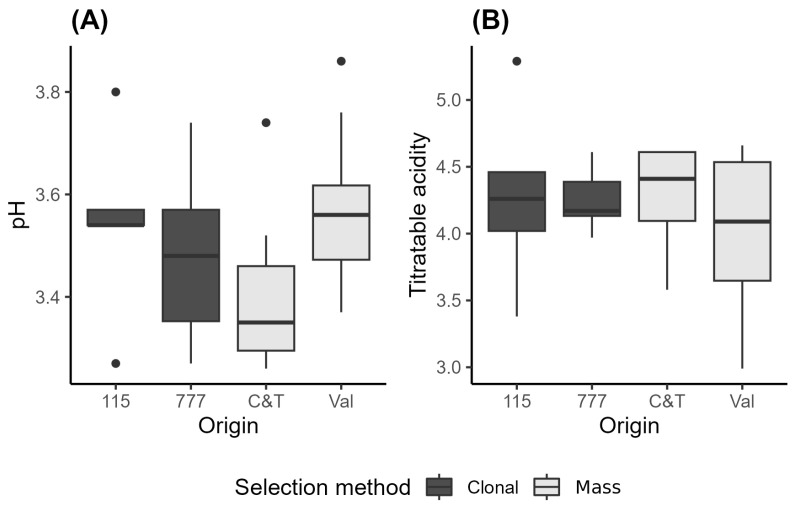
pH (**A**) and total acidity (g/L H_2_SO_4_) (**B**) determined in Pinot Noir wines from different selection methods. Clonal: 115 (*n* = 5) and 777 (*n* = 6); and mass selection: C&T (*n* = 7) and Val (*n* = 8). The boxes show the interquartile range (IQR), the horizontal line within each box indicates the median, and the whiskers extend to 1.5 × IQR.

Figure 1 shows the pH and total acidity of the wines from the Pinot Noir clones 115 and 777 and mass selections Val and C&T in a box plot. The averaged values and standard deviations for both analyses are also reported in Table S2.

The results of the current study show that the pH of the wines ranged from 3.41 ± 0.17 to 3.57 ± 0.17, with titratable acidity ranging from 4.00 ± 0.63 to 4.29 ± 0.39 g H_2_SO_4_/L. Although no statistically significant differences were observed among treatments, the full range of values was covered by wines from the mass selections (Figure 1).

In comparison, Casassa et al. (2024) [26] reported pH values of 3.56 ± 0.01 and 3.62 ± 0.02 for wines from the Pinot Noir clones UCD23 and 828, respectively, and total acidity values of 5.04 ± 0.09 and 4.96 ± 0.13 g H_2_SO_4_/L [26]. These pH values are slightly higher, and total acidity is notably higher than the values observed in the current study.

Additionally, Casassa et al. (2021) [27] observed pH values of 3.58 ± 0.02 and 3.56 ± 0.01 for wines from clones 115 and 777, respectively, with total acidity values of 3.48 ± 0.05 and 4.13 ± 0.13 g H_2_SO_4_/L. The pH values are comparable, while the total acidity is lower than those observed in the current study, except for the wines from clone 777, which are closer in range.

### 3.2. Global Phenolic Composition and Color of the Wines

**Figure 2:**
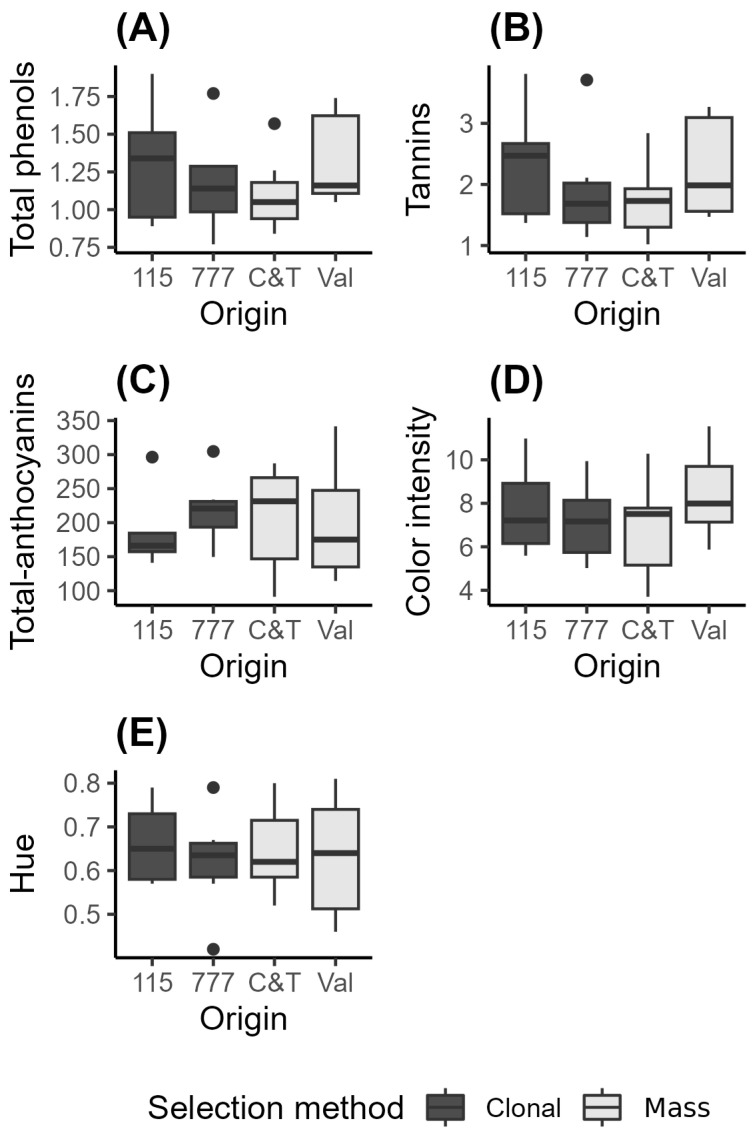
Total phenols (g/L gallic acid equivalents) (**A**), tannins (g/L catechin equivalents) (**B**), anthocyanins (mg/L malvidin-3-glucoside equivalents) (**C**), color intensity (a.u. 420 + 520 + 620 nm) (**D**), and hue (a.u. 420/520 nm) (**E**) determined in Pinot Noir wines from different selection methods. Clonal: 115 (*n* = 5) and 777 (*n* = 6); and mass selection: C&T (*n* = 7) and Val (*n* = 8). The boxes show the interquartile range (IQR), the horizontal line within each box indicates the median, and the whiskers extend to 1.5 × IQR.

Total phenols are a group of compounds found in wine that play a key role in its flavor, color, mouthfeel, and overall sensory profile. In red wines, including Pinot Noir, phenols primarily come from the grape skins, seeds, and stems, and they include anthocyanins, tannins, flavonols, and other phenolic compounds. The concentration of total phenols in Pinot Noir wines can vary significantly depending on factors such as grapevine genotype, viticultural practices, climate, and winemaking techniques [26,27]. Previous studies have reported values for Pinot Noir clones 115, 777, and 2A of 0.99, 1.18, and 1.04 g/L of catechin equivalents, respectively [27], which are comparable to the concentrations observed in this study: clone 115 (1.32 ± 0.42), clone 777 (1.18 ± 0.35), Val (1.32 ± 0.3), and C&T (1.10 ± 0.25) (Figure 2 and Table S3).

Total tannins in Pinot Noir wines are key phenolic compounds that significantly influence the wine’s structural integrity, mouthfeel, bitterness, and astringency [18]. A comprehensive study by Giglio et al. (2023) [28] analyzed 155 Pinot Noir wines from five New Zealand regions across vintages from 2009 to 2021, reporting total tannin concentrations ranging from 0.336 to 1.697 g/L [28]. In our current study, total tannin concentrations (g/L) are notably higher, particularly in clone 115 (2.37 ± 0.99), clone 777 (1.94 ± 1.84), Val (2.22 ± 0.76), and C&T (1.72 ± 0.62) (Figure 2), placing them at the upper end—or above—the range reported by Giglio et al. (2023) [28].

These concentrations are higher than those reported by Casassa et al. (2021) [27] for clones 115 (170.0 mg/L) and 777 (198.7 mg/L) [24], which may be due to differences in the methods used to analyze the total tannins [20]. It should be noted that the tannin concentrations reported here reflect the use of the Bate–Smith assay, a method that produces systematically higher values than precipitation-based assays (e.g., MCP or BSA) due to its broader reactivity toward polymeric flavan-3-ols and anthocyanidin-forming structures. This methodological behavior has been well documented, including comparative studies showing that Bate–Smith yields several-fold higher values than MCP/BSA when applied to Chilean wines [20].

Total anthocyanins in Pinot Noir wines are a key group of phenolic compounds that contribute to the wine’s color, particularly its red and purple nuances [12,13]. These pigments are primarily found in the grape skins and are responsible for the characteristic deep, vibrant color of red wines, including Pinot Noir [12]. According to Casassa et al. (2021) [27], wines made from clone 115 had relatively lower total anthocyanin concentrations [measured spectrophotometrically] compared to wines from clones 2A and 777, with values of 179.3, 256.8, and 265.6 mg/L, respectively. In this study, a considerable degree of variability was observed in total anthocyanin concentrations among wines from both clonal and mass selections. Mean concentrations (mg/L) ranged from 189.09 ± 61.99 in clone 115 to 219.13 ± 52.06 in clone 777, with intermediate values for the mass selections Val (201.79 ± 88.90) and C&T (205.07 ± 76.18). However, these differences were not statistically significant. These values are consistent with those reported by Picardo et al. (2019) [29] for Pinot Noir wines from Uruguay, which ranged from 209  ±  8 to 311  ±  21 mg/L, depending on the vinification method.

Color intensity refers to the depth or richness of the wine’s color, which is influenced by the total concentration of anthocyanins, their extraction during fermentation, and the wine’s exposure to oxygen and other environmental factors [12]. Pinot Noir, known for its relatively thin-skinned grapes, typically produces wines with lower color intensity compared to other red varieties, such as Cabernet Sauvignon or Syrah [27,28,29]. Hue, on the other hand, refers to the specific color tone of the wine, ranging from purple to ruby to brick red as the wine matures [12]. The hue of Pinot Noir wines is typically more delicate and lighter than that of other red wines, often showing a vibrant, bright red or purple coloration when young. Over time, as wine ages, the anthocyanins undergo chemical changes that lead to a shift in hue [12,26].

Regarding color intensity, the average values are lower than those described by other authors [27,30], with the following values: clone 115 (7.77 ± 2.2), clone 777 (7.17 ± 1.85), Val (8.43 ± 1.92), and C&T (6.77 ± 2.24), as shown in Figure 2.

Slightly lower values for hue were observed in the wines from both clones and mass selections [clone 115 (0.66 ± 0.1), clone 777 (0.62 ± 0.12), Val (0.63 ± 0.14), and C&T (0.65 ± 0.11)], compared to Pinot Noir wines from various regions of New Zealand [27]. These values are also similar to those found in wines from clones 115, 777, and 2A in California’s Central Coast [27]. Additionally, the values observed in this study fall within the range reported for different Pinot Noir wines subjected to various leaf removal treatments, with values ranging from 0.69 ± 0.01 to 0.86 ± 0.06 [31].

### 3.3. Fractions of Proanthocyanidins of Wines

Proanthocyanidins (PAs) are a type of flavonoid commonly found in wine, particularly in red wines like Pinot Noir. These compounds exist in different polymerization forms—monomeric, oligomeric, and polymeric—each contributing uniquely to the sensory and chemical properties of the wine, such as astringency, bitterness, and color stability [20].

Monomeric PAs are the simplest form, consisting of individual flavan-3-ol units, typically catechins and gallocatechins. They are primarily responsible for the astringency and bitterness of wine. Oligomeric PAs consist of a small number (2–10) of flavan-3-ol units and contribute significantly to astringency, as they tend to bind proteins more effectively than monomers. These compounds are often associated with the structure and mouthfeel of the wine. In this study, oligomeric proanthocyanidins (PAs) were defined as flavan-3-ol polymers containing approximately 2–10 monomer units, following conventions widely applied in chromatographic and depolymerization studies of red wine tannins [19,20]. Compounds exceeding this degree of polymerization were classified as polymeric PAs. We note that alternative classification schemes exist—such as the small and large polymeric pigment (SPP/LPP) system derived from the Harbertson–Adams assay—which describe pigment–tannin reaction products rather than native PA size distributions. Given the analytical resolution of the methods employed here and the focus on the intrinsic PA fractions, the oligomeric/polymeric framework represents the most appropriate and widely adopted approach for interpreting these data. Polymeric PAs are larger molecules formed by the polymerization of flavan-3-ol units. They are known for their long-lasting astringency and play an important role in the aging potential of wine. Polymeric PAs also contribute to the color stability of red wines, especially during storage, due to their ability to interact with anthocyanins and other phenolic compounds [20]. Fulcrand et al. (2006) demonstrated that polymeric proanthocyanidins are present in Pinot Noir wines and help maintain oxidative stability during aging [32].

The concentration of proanthocyanidins in wine is primarily determined by the grape’s proanthocyanidin content, as well as factors such as extraction methods, winemaking techniques, and aging conditions. The structural features of proanthocyanidins—such as composition, degree of polymerization, galloylation, and cis/trans ratio—also influence their concentration and characteristics in wine [33].

**Figure 3:**
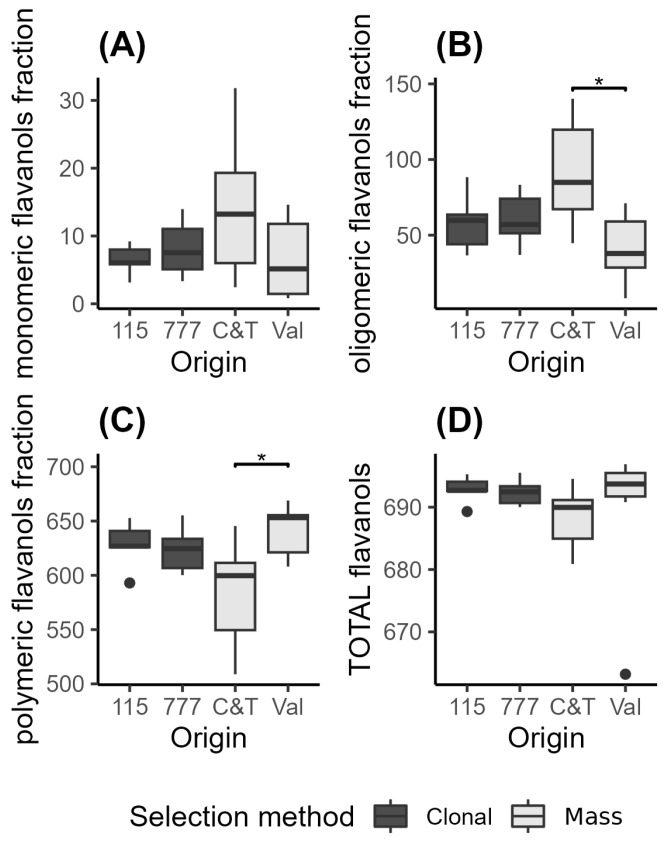
Fractions of monomeric (**A**), oligomeric (**B**), and polymeric flavanols (mg/L of catechin equivalents) (**C**) and total flavanols (**D**) of Pinot Noir wines from different selection methods. Clonal: 115 (*n* = 5) and 777 (*n* = 6); and mass selection: C&T (*n* = 7) and Val (*n* = 8). The boxes show the interquartile range (IQR), the horizontal line within each box indicates the median, and the whiskers extend to 1.5 × IQR. Horizontal brackets indicate statistically significant differences between means based on a Kruskal–Wallis test followed by a post hoc Dunn’s test (*p* < 0.05).

The distribution of monomeric, oligomeric, and polymeric proanthocyanidins observed in these wines (Figure 3), is consistent with patterns previously reported for *Vitis vinifera* varieties in general [33,34], which describe comparable proportions across cultivars. Further Pinot Noir–focused data (Carew et al., 2013) [35] also support the occurrence of similar PA distribution patterns in this variety.

When comparing the results of this study with those of Jordao et al. (2010) [34], who analyzed the monomeric, oligomeric, and polymeric fractions in 24 representative commercial Portuguese sparkling wines from the Bairrada Appellation of Origin, the average concentrations were 2.8 ± 0.56 mg/L for monomeric, 20.4 ± 1.12 mg/L for oligomeric, and 41.3 ± 1.97 mg/L for polymeric fractions. These values are lower than those observed for the wines from the two clones and two mass selections of Pinot Noir in this study (Figure 3 and Table S3). On the other hand, Monagas et al. (2003) [33] reported similar values for Tempranillo, Graciano, and Cabernet Sauvignon wines, except for the oligomeric fraction, where these varieties showed higher concentrations compared to the Pinot Noir wines in the present study.

Regarding the oligomeric and polymeric fractions, wines from both mass selections exhibit contrasting behaviors. The C&T selection shows higher levels of oligomers and lower levels of polymers, whereas the Val selection displays the opposite trend.

This observation aligns with findings from New Zealand Pinot Noir wines, where regional differences in tannin composition were noted. Specifically, wines from Martinborough exhibited higher concentrations of polymeric phenolics and tannins, which were associated with more robust mouthfeel attributes and a higher degree of tannin polymerization (mDP) [30].

The absence of correlation between total tannins and the distribution of proanthocyanidin size fractions (Figure S1) suggests that total tannin concentration alone is insufficient to describe tannin structural complexity in Pinot Noir wines. This observation points to more complex scenarios in which extraction dynamics, polymerization, depolymerization, and tannin–anthocyanin interactions may play a dominant role in shaping tannin refinement reactions, independently of bulk tannin levels.

Similarly, research on wines produced from different clones has demonstrated that clone selection significantly influences tannin composition and concentration. For instance, Pinot Noir wines from the AM10/5 clone exhibited higher tannin concentrations and a greater proportion of polymeric pigments compared to those from the UCD5 clone [31].

These findings underscore the importance of both mass selection and clone selection in determining the phenolic profile of Pinot Noir wines, which in turn affects their sensory characteristics and aging potential [28,30].

### 3.4. Individual Anthocyanins and Low Molecular Weight Phenolic Compounds of the Wines

This section discusses the content of individual anthocyanins and low molecular weight phenolic compounds, as determined by HPLC-DAD analyses, with values presented as mean ± standard deviation (in mg/L). A total of 18 compounds were quantified. These compounds are well-known as key constituents of red wines and were specifically chosen for their high abundance in Pinot Noir wines, as reported in previous studies [28,30].

**Figure 4:**
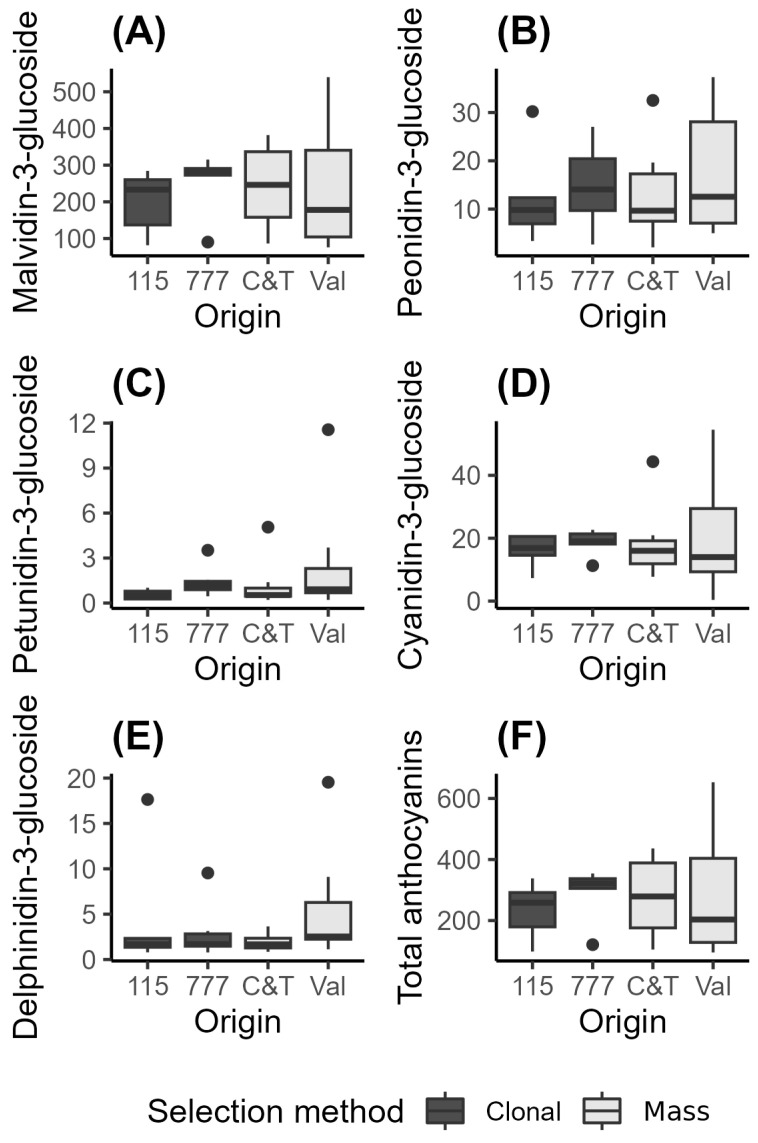
Individual anthocyanins quantified by HPLC-DAD in Pinot Noir wines obtained from different selection methods. Clonal selections: 115 (*n* = 5) and 777 (*n* = 6); mass selections: C&T (*n* = 7) and Val (*n* = 8). Boxplots represent the distribution of anthocyanin concentrations for each group. Boxes indicate the interquartile range (IQR), horizontal lines within boxes denote the median, and whiskers extend to 1.5 × IQR. Individual data points beyond the whiskers are shown as outliers. (**A**) Malvidin-3-glucoside; (**B**) Peonidin-3-glucoside; (**C**) Petunidin-3-glucoside; (**D**) Cyanidin-3-glucoside; (**E**) Delphinidin-3-glucoside; (**F**) Total anthocyanins.

**Figure 5:**
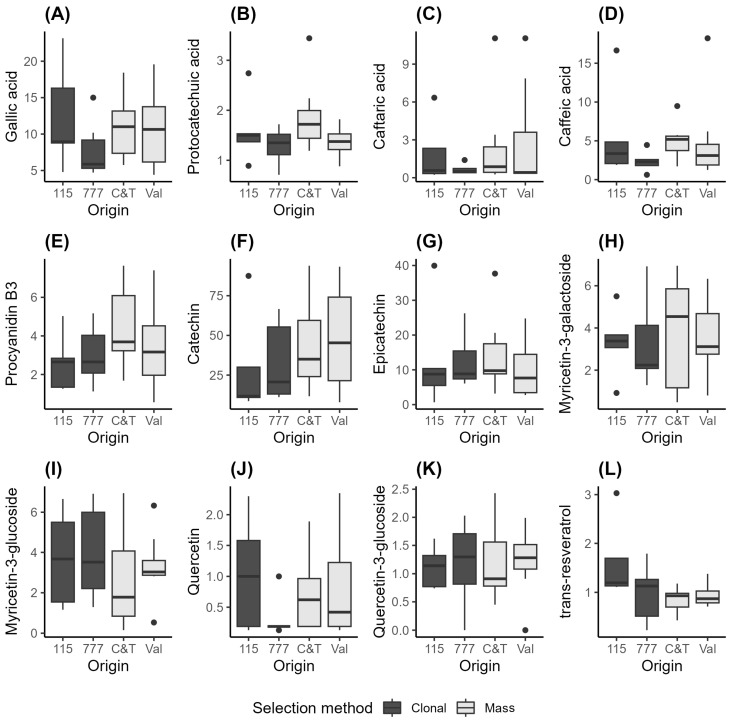
Individual flavan-3-ols quantified by HPLC-DAD in Pinot Noir wines obtained from different selection methods. Clonal selections: 115 (*n* = 5) and 777 (*n* = 6); mass selections: C&T (*n* = 7) and Val (*n* = 8). Boxplots represent the distribution of flavanol concentrations for each group. Boxes indicate the interquartile range (IQR), horizontal lines within boxes denote the median, and whiskers extend to 1.5 × IQR. Individual data points beyond the whiskers are shown as outliers. (**A**) Gallic acid; (**B**) Protocatechuic acid; (**C**) Caftaric acid; (**D**); Caffeic acid (**E**) Procyanidin B3; (**F**) Catechin; (**G**) Epicatechin; (**H**) Myricetin-3-galactoside; (**I**) Myricetin-3-glucoside; (**J**) Quercetin; (**K**) Quercetin-3-glucoside; (**L**) trans-resveratrol.

The data for these parameters are graphically summarized in Figure 4 and Figure 5 and Table S4, which facilitate a comparison of the data spread for the two clones and two mass selections. To compare the parameters, the scale of expression of the values can be considered. Kruskal–Wallis tests were conducted to assess any significant differences between the parameters measured for the two clones and the two mass selections. For the discussion, the compounds have been grouped into their primary classes: anthocyanins, phenolic acids, catechins, flavonols, and stilbenes.

#### 3.4.1. Individual Anthocyanins

Pinot Noir, Pinot Meunier, and Pinot Madeleine grapes do not synthesize acetylated anthocyanins [35]. Because of this, Pinot Noir grapes typically exhibit a simpler anthocyanin profile, with five primary anthocyanins being most abundant: malvidin-3-glucoside, petunidin-3-glucoside, peonidin-3-glucoside, delphinidin-3-glucoside, and cyanidin-3-glucoside. Among these, malvidin-3-glucoside is usually the most prevalent, followed by peonidin and petunidin glucosides. The five anthocyanins identified in Pinot Noir are the result of a less complex biosynthesis pathway in comparison to other red wine grapes like Cabernet Sauvignon. This contributes to a lighter, more translucent wine color and a less intense red hue in Pinot Noir, which is characteristic of this variety [36].

Malvidin-3-glucoside was by far the most abundant anthocyanin in the wines from the two clones and two mass selections, representing approximately 85% of the total anthocyanin concentration. The observed values for the wine samples were as follows: clone 115 (199.33 ± 86.38), clone 777 (255.59 ± 82.39), Val (246.33 ± 185.88), and C&T (243.38 ± 115.41). Notably, there was high dispersion in the total anthocyanin levels and the concentration of malvidin-3-glucoside (Figure 4), with the Val mass selection showing a higher variability, in contrast to the more consistent results observed in the wines from clone 777.

These findings align with previous studies indicating that anthocyanin composition and concentration can vary significantly among different clones of the same grape variety.

Studies on Pinot Noir clones have shown that certain clones, such as AM10/5, produce wines with higher anthocyanin concentrations and a greater proportion of polymeric pigments compared to others like UCD5 [35]. This suggests that the genetic makeup of each clone plays a crucial role in determining the anthocyanin profile and, consequently, the color and aging potential of the wine.

#### 3.4.2. Phenolic Acids

As previously reported by other authors, gallic, caffeic, and caftaric acids are typically the most abundant acids in red wines, although their ratios can vary [28,37]. The ranges of all these compounds are shown in Figure 5.

The predominance of gallic acid as the most abundant phenolic acid in Pinot Noir wines is consistent with earlier studies that examined the phenolic profiles of red wines. In our study, gallic acid concentrations were highest across all evaluated clones and mass selections, notably in clone 115 (12.39 ± 7.31 mg/L) and Val (10.71 ± 5.28 mg/L). This pattern aligns with findings by Casassa et al. (2024) [26], who reported similarly high levels of gallic acid in Pinot Noir wines subjected to reduced cap management. Their results, obtained at an industrial scale, support the role of the clone and vinification approach in shaping phenolic acid content [26].

Caffeic acid was the second most abundant acid, surpassing its esterified derivative, caftaric acid. Our observed caffeic acid concentrations were notably higher in clone 115 (5.76 ± 6.2 mg/L) and mass selection C&T (5.01 ± 2.42 mg/L), while caftaric acid remained lower across all samples. These findings echo the observations by Casassa et al. (2021) [27], who also reported caffeic acid as a more dominant compound than caftaric acid when evaluating extended maceration and saignée treatments in Pinot Noir vinifications. Their work highlights that processing techniques, such as stem addition and maceration duration, can influence the hydrolysis of caftaric acid to caffeic acid, especially under enzymatic or oxidative conditions [27].

Protocatechuic acid was the least abundant among the phenolic acids measured, a trend consistent with several previous characterizations of Pinot Noir. For example, Giglio et al. (2023) [28] quantified phenolic acids in New Zealand Pinot Noir wines and similarly identified low concentrations of protocatechuic acid compared to gallic and caffeic acids. Their chemometric analyses suggested that while protocatechuic acid contributes to the antioxidant properties of wine, it plays a less significant role in defining chromatic or sensory profiles due to its lower abundance [28].

In a broader regional and compositional context, Yang et al. (2025) [30] also documented that phenolic acid levels, especially gallic and caffeic acids, vary significantly depending on clone, region, and wine matrix interactions in New Zealand Pinot Noir wines. Their findings reinforce the relevance of clone-specific expression and environmental modulation of phenolic acid biosynthesis and extraction in final wine composition [30].

#### 3.4.3. Catechins-Procyanidins

Four flavan-3-ols were analyzed in this study. In terms of abundance, (+)-catechin was the most prevalent, followed by (−)-epicatechin, epicatechin gallate, and procyanidin B1. The ranges of all these compounds are shown in Figure 5 and in Table S4.

(+)-Catechin was the most abundant flavan-3-ol in both clones and mass selections: clone 115 (40.71 ± 30.04), clone 777 (38.95 ± 30.59), Val (40.39 ± 30.74), and C&T (43.57 ± 29.06). (−)-Epicatechin was present at lower concentrations compared to (+)-catechin: clone 115 (14.40 ± 11.64), clone 777 (14.80 ± 12.09), Val (15.75 ± 12.08), and C&T (14.97 ± 12.31).

These values were lower than those reported by Giglio et al. (2023) for a set of 155 Pinot Noir wines from New Zealand [28], where (+)-catechin ranged from 60 to 239 mg/L and (−)-epicatechin ranged from 19 to 106 mg/L. Additionally, the values were lower than those described by Yang et al. (2025) [30] for 116 Pinot Noir wines from various regions of New Zealand [30], with (+)-catechin ranging from 119.02 ± 38.73 to 145.50 ± 37.71 mg/L and (−)-epicatechin ranging from 47.68 ± 15.88 to 60.71 ± 18.96 mg/L.

Our study revealed that epicatechin gallate and the dimer procyanidin B1 were present at notably lower concentrations than monomeric flavan-3-ols such as (+)-catechin and (−)-epicatechin across all clones and mass selections. Procyanidin B1 ranged from 4.18 ± 4.60 mg/L in clone 115 to 4.80 ± 5.10 mg/L in C&T, while epicatechin gallate ranged from 3.31 ± 2.41 mg/L (clone 777) to 6.10 ± 6.58 mg/L (C&T). These patterns are consistent with the findings by Fortes-Gris et al. (2011) [38], who reported that polymeric procyanidins (dimers) and galloylated flavan-3-ols were present in smaller amounts than monomeric catechins in wines from different red varieties.

#### 3.4.4. Flavonols

The flavonol composition of the wines is shown in Figure 5. Flavonols are significant phenolic constituents in wine, contributing to color enhancement and stabilization through copigmentation interactions with anthocyanins. Moreover, flavonols have been associated with modulating astringency perception, particularly imparting a velvety mouthfeel [26]. Flavonol glucosides are primarily found in grape skins, and their concentration can be influenced by factors such as grapevine variety and environmental conditions. During fermentation, flavonoid glucosides, like quercetin-3-glucoside, can be hydrolyzed into free flavonols, such as quercetin, which then contribute to the wine’s final flavonol profile [39].

In the analyzed Pinot Noir samples, three flavonol glucosides—myricetin-3-glucoside, myricetin-3-galactoside, and quercetin-3-glucoside—along with the aglycone quercetin, were identified and quantified. The concentration levels of these compounds showed minimal variation across the different clones and mass selections studied. Overall, the measured concentrations (ranging from 0.32 ± 0.34 to 3.97 ± 2.39 mg/L) were lower than those previously reported for Pinot Noir wines in the literature [28,40].

#### 3.4.5. Stilbenes

Stilbenes, such as trans-resveratrol, are a group of polyphenolic compounds found in wine, particularly in red wine varieties like Pinot Noir. During the grape ripening process, trans-resveratrol is produced as part of the plant’s defense mechanism against biotic and abiotic stress produced by fungal infections, UV radiation, or mechanical injury. In general, wines made from grape varieties with thicker skins, such as Pinot Noir, tend to have higher concentrations of trans-resveratrol compared to wines made from thinner-skinned varieties. Additionally, wines produced in cooler climates often have higher trans-resveratrol concentrations, as the plant’s defense mechanisms against environmental stressors are more activated [41,42].

In this study, the concentrations of trans-resveratrol for the two clones and two mass selections were as follows: clone 115 (1.63 ± 0.93), clone 777 (0.90 ± 0.83), Val (0.87 ± 0.38), and C&T (0.89 ± 0.39). These values are lower than those reported by Giglio et al. (2023) [28] and Yang et al. (2025) [30], but are similar to or higher than those found in Burgundy Pinot Noir wines by Jeandet et al. (1993) [43].

### 3.5. Wine Compositions According to Clonal or Mass Groups

**Figure 6:**
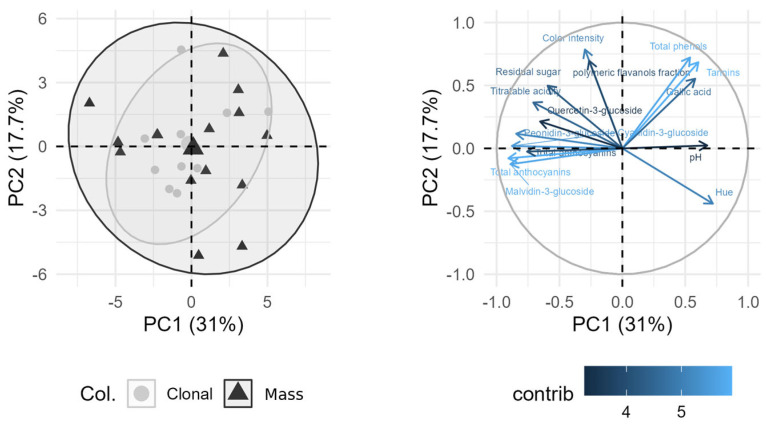
Principal component analysis (PCA) of Pinot Noir samples, displaying the first two principal components (PC1 and PC2), which explain 31% and 17.7% of the total variance, respectively. (**Left**) Score plot of individual samples: circles represent Clonal selections, and triangles represent Masal selections. Ellipses denote the unit radius in the PCA space. (**Right**) Loading plot (variable factor map) illustrating the top 15 contributing variables as arrows, color-coded according to their contribution to the principal components (from lower to higher). The outer circle indicates the correlation circle (radius = 1), where variables positioned farther from the origin have stronger correlations with the principal components.

A PCA (Principal Component Analysis) biplot (Figure 6) was used to visualize wine chemical composition according to type of group (clonal or mass). For PCA visualization, the 15 variables with the highest contribution scores were retained to maximize the proportion of variance explained while ensuring orthogonality and maintaining a manageable, interpretable set of loadings. The first two PCs explained 48.7% of the variance among the sample set.

Clonal wines clustered on the right side of the plot, associated with higher levels of individual and total anthocyanins, color intensity, (−)-epicatechin, quercetin-3-glucoside and total acidity. Mass wines displayed a diverse chemical profile, with the majority positioned on the left side of the plot, overlapping some chemical characteristics with clonal wines. Mass wines clustered more towards the left side of the plot in association with total tannin, (+)-catechin, procyanidin B1, gallic, protocatechuic and caffeic acids, protocatechuic pH and hue. The present study reveals a high degree of variability in several chemical parameters across the different groups of samples corresponding to each clone or mass selection. This finding is consistent with previous research by Miele (2021) [44], who investigated the wine composition of four Merlot and five Cabernet Sauvignon clones under the same environmental conditions in Serra Gaúcha, Brazil. Miele observed that vintage had a much stronger influence on wine composition than the clone itself, with no significant differences found in parameters such as total phenols, tannins, anthocyanins, or color intensity. Similarly, Martin et al. (2022) [45] suggested that the influence of berry weight on wine composition may outweigh the individual effects of vintage, region, vineyard, or vine yield.

However, contrasting results were reported by Casassa et al. (2021) [27] in their study of the phenolic composition of three Pinot Noir clones. They found that clones with higher berry fresh mass at harvest—such as clones 2A (1.12 ± 0.02 g) and 115 (1.10 ± 0.01 g)—compared to clone 777 (0.99 ± 0.05 g), had lower concentrations of total berry anthocyanins but similar levels of total phenols.

These results underscore the complex and sometimes divergent effects of clonal and mass selection on the phenolic composition of grapes and wines, indicating that while certain factors may enhance consistency, others may result in marked variability.

The overarching outcome of this survey was the marked chemical homogeneity observed among the commercial Pinot Noir wines, regardless of plant material origin. This pattern indicates that phenolic expression under Chilean conditions is highly context-dependent and largely shaped by winemaking and vineyard environment rather than by clonal or mass-selection identity.

### 3.6. Preliminary Evaluation of Regional Effects on Wine Composition

Given the small and uneven sample size across PDOs, this analysis was restricted to a descriptive overview. Considering the observational nature of the dataset, PCA represents a conservative and statistically sound choice for exploring compositional patterns, whereas supervised alternatives such as OPLS-DA would risk overinterpretation.

**Figure 7:**
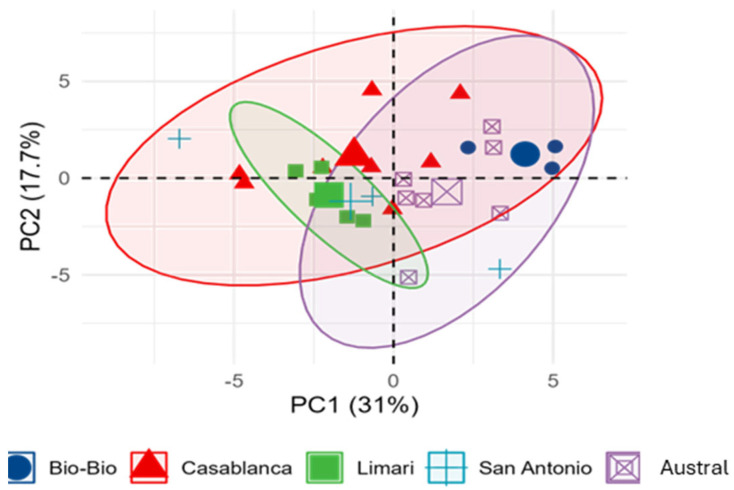
PCA biplot of wine samples by region with loadings of chemical composition variables. The first two principal components (PC1 and PC2) explain 31.0% and 17.7% of the total variance, respectively.

No consistent regional trends were observed for basic chemical parameters, total phenolics, anthocyanins, or tannin-derived fractions. The substantial overlap among regions suggests that, within this commercial dataset, geographic origin does not emerge as a primary source of compositional variation (Figure 7). However, the limited statistical power associated with the sample distribution prevents definitive conclusions. More balanced and controlled studies are necessary to assess regional influences on Pinot Noir composition.

### 3.7. Implications for Viticulture and Winemaking

The limited compositional differentiation among wines produced from distinct Pinot Noir clones and mass selections indicates that, under Chilean commercial conditions, phenolic expression is predominantly shaped by environmental and winemaking factors rather than by plant material. Consequently, the use of certified clones may be most advantageous for agronomic attributes rather than for generating distinct phenolic profiles, while traditional mass selections remain suitable planting materials. These findings highlight that terroir-driven factors—such as soil type, mesoclimate, and canopy architecture—as well as extraction and fermentation management exert a stronger influence on wine composition than clonal differences. Thus, producers seeking stylistic differentiation in Chilean Pinot Noir may achieve greater impact through vineyard and cellar practices than through modifications in plant material.

## 4. Conclusions

In conclusion, this preliminary study evaluated, for the first time to our knowledge, the chemical composition of wines made at an industrial scale from two mass selections and two clones of Pinot Noir cultivated in Chile. While some variation was observed in certain spectrophotometric parameters, differences were not consistent across all phenolic variables. No clear trends emerged in the concentrations of procyanidins, individual phenolic compounds, or other key wine parameters among the selections analyzed. These findings suggest that, under commercial winemaking conditions and in the absence of standardized viticultural and enological practices, the influence of grapevine genotype—whether clonal or massal—on wine phenolic composition appears to be modest and context-dependent. Further studies under controlled conditions are needed to more accurately assess the role of plant material in shaping the chemical and sensory attributes of Pinot Noir wines.

## Data Availability

The original contributions presented in this study are included in the article/Supplementary Material. Further inquiries can be directed to the corresponding author (A.P.-N.). The raw data supporting the conclusions of this article will be made available by the authors on request.

## Supplementary Materials

The following supporting information can be downloaded at: https://www.mdpi.com/article/10.3390/plants15030359/s1, Table S1. Summary of key edaphic descriptors of the Chilean viticultural zones represented in the Pinot Noir wines included in this study. Table S2. Mean values and standard deviations (*n* = 26) for the residual sugars, total acidity, pH and color parameters (intensity and hue) of Pinot noir wines from different clones and mass se-lections. Different letters in a row indicate statistical differences (*p* < 0.05) according to a non-parametric Kruskal-Wallis test. Table S3. Mean values and standard deviations (*n* = 26) for the total phenols (g/L of gallic acid equivalents), tannins (g/L of catechin equivalents) and anthocyanins (mg/L of malvidin-3-glucoside equivalents), and the fractions of monomeric, oligomeric, and polymeric proanthocyanidins (PA) (mg/L of catechin equivalents) of Pinot noir wines from different clones and mass selections. Different letters in a row indicate statistical differences (*p* < 0.05) according to a non-parametric Kruskal-Wallis test. Table S4. Mean values of concentration (mg/L) and standard deviations (*n* = 26) of the individual polyphenolic compounds belonged to different chemical families identified by HPLC-DAD in Pinot noir red wines from different clones and mass selections. Different letters in a row indicate statistical differences (*p* < 0.05) according to a non-parametric Kruskal-Wallis test. Table S5. Mean values of concentration (mg/L) and standard deviations (*n* = 26) of the individual polyphenolic compounds belonging to different chemical families identified by HPLC-DAD in Pinot Noir red wines from different origin Valey. Figure S1. Relationship between total tannin concentration and the different proanthocyanidin (PA) fractions (monomeric, oligomeric, and polymeric) in commercial Chilean Pinot Noir wines. Scatter plots show the absence of a linear correlation between total tannins and individual PA fractions (r ≈ 0 in all cases). This lack of association suggests that total tannin content does not directly reflect the distribution of PA size classes, highlighting the presence of more complex and possibly independent mechanisms governing tannin polymerization, transformation, and refinement reactions during winemaking and wine aging.

## References

[1] International Organization of Vine and Wine (2017). Distribution of the World’s Grapevine Varieties.

[2] Robinson J., Harding J., Vouillamoz J. (2012). Wine Grapes: A Complete Guide to 1368 Vine Varieties, Including Their Origins and Flavours.

[3] Grainger K., Tattersall H. (2016). Wine Production and Quality.

[4] Castagnoli S.P., Vasconcelos M.C. (2006). Field Performance of 20 ‘Pinot Noir’ Clones in the Willamette Valley of Oregon. HortTechnology.

[5] Collas A., Barillère J.-M., Bougerey C., Palgé C. Clonal Selection in Champagne. Proceedings of the International Symposium on Clonal Selection.

[6] Bernard R. Aspects of Clonal Selection in Burgundy. Proceedings of the International Symposium on Clonal Selection.

[7] Blaich R., Konradi J., Rühl E., Forneck A. (2007). Assessing Genetic Variation among Pinot Noir (*Vitis vinifera* L.) Clones with AFLP Markers. Am. J. Enol. Vitic..

[8] Knowles T., Sharples L. (2002). The History and Development of Chilean Wines. Int. J. Wine Mark..

[9] Rojas L.M. (1897). Tratado de Viticultura y Vinificación.

[10] Servicio Agrícola y Ganadero (SAG), Ministerio de Agricultura de Chile (2025). Sección Viñas y Vinos de la División de Protección Agrícola y Forestal. https://www.sag.gob.cl/ambitos-de-accion/vinas-y-vinos.

[11] Meneses M., Castro M.H., Hinrichsen P. (2024). Genetic Characterization of Criolla and European Grape-vines Recently Found in Chile: A Key Step for Their Rescue and Conservation. Aust. J. Grape Wine Res..

[12] Ribéreau-Gayon P., Glories Y., Maujean A., Dubourdieu D., Ribéreau-Gayon P. (2006). Phenolic Compounds. Handbook of Enology.

[13] Duley G., Dujourdy L., Klein S., Werwein A., Spartz C., Gougeon R.D., Taylor D.K. (2021). Regionality in Australian Pinot Noir Wines: A Study on the Use of NMR and ICP-MS on Commercial Wines. Food Chem..

[14] Roullier-Gall C., Boutegrabet L., Gougeon R.D., Schmitt-Kopplin P. (2014). A Grape and Wine Chemodiversity Comparison of Different Appellations in Burgundy: Vintage vs Terroir Effects. Food Chem..

[15] Burin V.M., Freitas Costa L., Rosier J.P., Bordignon-Luiz M.T. (2011). Cabernet Sauvignon wines from two different clones, characterization and evolution during bottle ageing. LWT-Food Sci. Technol..

[16] Luzio W. (2010). Suelos de Chile.

[17] International Organisation of Vine and Wine (2016). Compendium of International Methods of Wine and Must Analysis.

[18] Bate-Smith E. (1981). Astringent Tannins of the Leaves of Geranium Species. Phytochemistry.

[19] Sun B., Leandro C., Da Silva J.M., Spranger I. (1998). Separation of Grape and Wine Proanthocyanidins According to Their Degree of Polymerization. J. Agric. Food Chem..

[20] Cáceres-Mella A., Peña-Neira Á., Avilés-Gálvez P., Medel-Marabolí M., Del Barrio-Galán R., López-Solís R., Canals J.M. (2014). Phenolic Composition and Mouthfeel Characteristics Resulting from Blending Chilean Red Wines. J. Sci. Food Agric..

[21] Fanzone M., Peña-Neira Á., Gil M., Jofré V., Assof M., Zamora F. (2012). Impact of Phenolic and Polysaccharidic Composition on Commercial Value of Argentinean Malbec and Cabernet Sauvignon Wines. Food Res. Int..

[22] Peña-Neira Á., Cáceres A., Pastenes C. (2007). Low Molecular Weight Phenolic and Anthocyanin Composition of Grape Skins from cv. Syrah (*Vitis vinifera* L.) in the Maipo Valley (Chile): Effect of Clusters Thinning. Food Sci. Technol. Int..

[23] Kassambara A. (2023). rstatix: Pipe-Friendly Framework for Basic Statistical Tests. R Package Version 0.7.2. https://cran.r-project.org/web/packages/rstatix/index.html.

[24] Kassambara A., Mundt F. (2020). Factoextra: Extract and Visualize the Results of Multivariate Data Analyses (R Package Version 1.0.7). https://cran.r-project.org/web/packages/factoextra/index.html.

[25] Kassambara A. (2023). ggcorrplot: Visualization of a Correlation Matrix Using “ggplot2” (R Package Version 0.1.4.1). CRAN. https://cran.r-project.org/web/packages/ggcorrplot/readme/README.html.

[26] Casassa L.F., Kuster S., Perlette D.J., Bargetto K.L. (2024). Chemical and Chromatic Composition of Wines Produced with Reduced Cap Management. J. Food Comp. Anal..

[27] Casassa L.F., Vega-Osorno A.A., Hernandez J.P. (2021). Chemical and Chromatic Effects of Saignée Combined with Extended Maceration. Aust. J. Grape Wine Res..

[28] Giglio C., Yang Y., Kilmartin P. (2023). Analysis of Phenolics in New Zealand Pinot Noir Wines Using UV-Visible Spectroscopy and Chemometrics. J. Food Comp. Anal..

[29] Piccardo D., Favre G., Pascual O., Canals J.M., Zamora F., González-Neves G. (2019). Influence of the Use of Unripe Grapes to Reduce Ethanol Content. Eur. Food Res. Technol..

[30] Yang Y., Ye Z., Araujo L.D., Rutan T., Deed R.C., Kilmartin P.A. (2025). Inter-Regional Characterisation of New Zealand Pinot Noir Wines. Food Chem..

[31] Wimalasiri P.M., Harrison R., Donaldson I., Kemp B., Tian B. (2024). Timing of leaf removal modulates tannin composition and the level of anthocyanins and methoxypyrazines in Pinot noir grapes and wines. Food Res. Int..

[32] Fulcrand H., Morel-Salmi C., Mané C., Poncet-Lengrand C., Vernhet A., Chenier V. Tannins: From reactions to complex supramolecular structures. Proceedings of the Advances in Tannin and Tannin Management, Australian Society of Viticulture and Oenology (ASVO) Seminar.

[33] Monagas M., Gómez-Cordovés C., Bartolomé B., Laureano O., Da Silva J.M. (2003). Monomeric, Oligomeric, and Polymeric Flavan-3-ol Composition of Wines. J. Agric. Food Chem..

[34] Jordão A.M., Gonçalves F.J., Correia A.C., Cantão J., Rivero-Pérez M.D., González SanJosé M.L. (2010). Proanthocyanidin Content, Antioxidant Capacity and Scavenger Activity of Portuguese Sparkling Wines. J. Sci. Food Agric..

[35] Carew A., Smith P., Close D., Curtin C., Dambergs R. (2013). Yeast Effects on Pinot noir Wine Phenolics, Color, and Tannin Composition. J. Agric. Food Chem..

[36] Dimitrovska M., Bocevska M., Dimitrovski D., Murkovic M. (2011). Anthocyanin Composition of Vranec, Cabernet Sauvignon, Merlot and Pinot Noir Grapes. Eur. Food Res. Technol..

[37] Van Leeuw R., Kevers C., Pincemail J.-L., Defraigne J.-O., Dommes J. (2014). Antioxidant capacity and phenolic composition of red wines from various grape varieties: Specificity of Pinot Noir: Specificity of Pinot Noir. J. Food Compos. Anal..

[38] Gris E.F., Mattivi F., Ferreira E.A., Vrhovsek U., Pedrosa R.C., Bordignon-Luiz M.T. (2011). Proanthocyanidin Profile and Antioxidant Capacity of Brazilian *Vitis vinifera* Red Wines. Food Chem..

[39] Castillo-Muñoz N., Gómez-Alonso S., García-Romero E., Hermosín-Gutiérrez I. (2007). Flavonol Profiles of *Vitis vinifera* Red Grapes and Their Single-Cultivar Wines. J. Agric. Food Chem..

[40] Serni E., Pedri U., Valls J., Sanoll C., Dordevic N., Überegger E., Robatscher P. (2020). Chemical Description and Organoleptic Evaluation of Pinot Noir Wines from Different Parts of Italy. OENO One.

[41] Lattanzio V., Lattanzio V.M., Cardinali A. (2006). Role of phenolics in the resistance mechanisms of plants against fungal pathogens and insects. Phytochem. Adv. Res..

[42] Jeandet P., Bessis R., Sbaghi M., Meunier P., Trollat P. (1995). Resveratrol Content of Wines of Different Ages: Relationship with Fungal Disease Pressure in the Vineyard. Am. J. Enol. Vitic..

[43] Jeandet P., Bessis R., Maume B.F., Sbaghi M. (1993). Analysis of Resveratrol in Burgundy Wines. J. Wine Res..

[44] Miele A. (2021). Wine Composition of Merlot and Cabernet Sauvignon Vine Clones under the Environmental Conditions of Serra Gaúcha, Brazil. Food Sci. Technol..

[45] Martin D., Lindsay M., Kilmartin P., Dias Araujo L., Rutan T., Yvon M., Stuart L., Grab F., Moore T., Scofield C. (2022). Grape Berry Size Is a Key Factor in Determining New Zealand Pinot Noir Wine Composition. OENO One.

