# Supplementary material for: Chemical Profile and Phenolic Composition of Commercial Chilean Pinot Noir Wines from Clonal and Mass-Selection
Source: Plants (Basel). 2026 Jan 23;15(3):359. doi: 10.3390/plants15030359 (PMC12899682; doi:10.3390/plants15030359)
Supplement: Supplementary file 1 [file plants-15-00359-s001.zip › plants-3964071-supplementary.pdf]

Supplementary Materials.

Table S1. Summary of key edaphic descriptors of the Chilean viticultural zones represented in the Pinot Noir wines included in this study.

Region/ PDO	Coordinates (central reference)	Soil Origin & Parent Material	Typical Soil Texture	Soil Hydrology & Chemical Properties
Limarí (LR)	30°37′56″ S, 71°18′51″ W	Alluvial–colluvial	Clay to clay-loam over loamy subsoil with gravel	Well-drained; low–moderate water retention; moderately alkaline; $CaCO_3$ accumulation
Casablanca (CB)	33°19′12″ S, 71°25′12″ W	soils over calcareous marine sediments	Sandy loam to loam; shallow profiles	Well drained; low water-holding capacity; low organic matter; moderate fertility
Leyda–San Antonio (LSA)	33°36′00″ S, 71°28′00″ W	Granitic parent material under alluvial influence	Sandy loam	Well drained; moderately acidic; enhanced mineral weathering
Biobío (S)	37°43′08″ S – 38°15′00″ S / 72°14′27″ W – 72°40′00″ W	Decomposed granitic soils, highly weathered	Clay to clay-loam	High water retention; slow drainage; acidic; rich in Fe and Al oxides
Austral (A)	40°34′12″ S, 73°07′48″ W	Volcanic ash and pyroclastic deposits	Deep loamy Andisols to poorly drained Ñadis	Andisols: high organic matter, high moisture retention, acidic; Ñadis: poor drainage, seasonal saturation

Table S2. Mean values and standard deviations (n=26) for the residual sugars, total acidity, pH and color parameters (intensity and hue) of Pinot noir wines from different clones and mass selections. Different letters in a row indicate statistical differences ($p<0.05$) according to a non-parametric Kruskal-Wallis test.

**Analysis**	**Clon 115 (n=5)**	**Clon 777 (n=6)**	**Massal Val (n=7)**	**Massal C&T (n=8)**
Residual sugars[1]	3.90 ± 0.31 a	3.85 ± 0.06 a	3.78 ± 0.24 a	3.85 ± 0.12 a
Total acidity[2]	4.28 ± 0.69 a	4.25 ± 0.24 a	4.00 ± 0.63 a	4.29 ± 0.39 a
Alcohol content[3]	13.70 ± 0.40 a	13.60 ± 0.40 a	13.50 ± 0.40 a	13.60 ± 0.50 a
pH	3.54 ± 0.19 a	3.48 ± 0.18 a	3.57 ± 0.17 a	3.41 ± 0.17 a
Color Intensity	7.77 ± 2.2 a	7.17 ± 1.85 a	8.43 ± 1.92 a	6.77 ± 2.24 a
Hue	0.66 ± 0.1 a	0.62 ± 0.12 a	0.63 ± 0.14 a	0.65 ± 0.11 a

[1] Reducing sugars expressed in g/L of glucose equivalents. [2.] Results expressed in g/l of H_2SO_4 equivalents.[3] % v/v.

Table S3. Mean values and standard deviations (n=26) for the total phenols (g/L of gallic acid equivalents), tannins (g/L of catechin equivalents) and anthocyanins (mg/L of malvidin-3-glucoside equivalents), and the fractions of monomeric, oligomeric, and polymeric proanthocyanidins (PA) (mg/L of catechin equivalents) of Pinot noir wines from different clones and mass selections. Different letters in a row indicate statistical differences ($p<0.05$) according to a non-parametric Kruskal-Wallis test.

**Phenolics**	**Clon 115 (n=5)**	**Clon 777 (n=6)**	**Massal Val (n=7)**	**Massal C&T (n=8)**
Total Phenols	1.32 ± 0.42 a	1.18 ± 0.35 a	1.32 ± 0.29 a	1.10 ± 0.25 a
Total Tannins	2.37 ± 0.99 a	1.94 ± 0.93 a	2.26 ± 0.79 a	1.72 ± 0.62 a
Anthocyanins	189.09 ± 61.99 a	219.13 ± 52.06 a	201.79 ± 88.9 a	205.07 ± 76.18 a
Monomeric	6.4 ± 5.5 a	8.1 ± 4.2 a	6.5 ± 5.5 a	14.0 ± 10.4 a
Oligomeric	58.5 ± 20.0 ab	60.6 ± 17.6 ab	41.4 ± 21.5 b	91.9 ± 36.3 a
Polymeric	627.9 ± 22.4 ab	623.7 ± 20.9 ab	642.3 ± 22.3 a	582.3 ± 47.7 b

Table S4. Mean values of concentration (mg/L) and standard deviations (n=26) of the individual polyphenolic compounds belonged to different chemical families identified by HPLC-DAD in Pinot noir red wines from different clones and mass selections. Different letters in a row indicate statistical differences ($p<0.05$) according to a non-parametric Kruskal-Wallis test.

Phenolic compound	Clon 115 (n=5)	Clon 777 (n=6)	Massal Val (n=7)	Massal C&T (n=8)
Glucosylated Anthocyanidins				
Delphinidin-3-glucoside	4.76 ± 7.22 a	3.05 ± 3.27 a	5.58 ± 6.19 a	1.92 ± 0.96 a
Cyanidin-3-glucoside	16.04 ± 5.54 a	18.66 ± 4.09 a	21.39 ± 19.1 a	18.6 ± 12.22 a
Petunidin-3-glucoside	0.56 ± 0.34 a	1.44 ± 1.08 a	2.56 ± 3.8 a	1.24 ± 1.72 a
Peonidin-3-glucoside	12.54 ± 10.42 a	14.77 ± 8.91 a	17.68 ± 13.16 a	13.4 ± 10.16 a
Malvidin-3-glucoside	199.33 ± 86.38 a	255.59 ± 82.39 a	246.33 ± 185.88 a	243.38 ± 115.41 a
Benzoic acids				
Gallic acid	12.39 ± 7.31 a	7.81 ± 4.03 a	10.71 ± 5.28 a	10.89 ± 4.46 a
Protocatechuic acid	1.61 ± 0.68 a	1.29 ± 0.37 a	1.37 ± 0.30 a	1.89 ± 0.76 a
Hydroxicynnamic acids and derivatives				
Caffeic acid	5.76 ± 6.2 a	2.33 ± 1.27 a	4.92 ± 5.61 a	5.01 ± 2.42 a
Caftaric acid	1.96 ± 2.59 a	0.66 ± 0.4 a	2.87 ± 4.2 a	2.56 ± 3.9 a
Stilbenes				
Trans-resveratrol	1.63 ± 0.93 a	0.90 ± 0.83 a	0.87 ± 0.38 a	0.89 ± 0.39 a
Flavan-3-ols				
(+)-Catechin	29.75 ± 33.39 a	32.76 ± 26.13 a	48.33 ± 34.47 a	43.95 ± 29 a
(-)-Epicatechin	13.05 ± 15.48 a	12.42 ± 7.88 a	10.2 ± 8.21 a	14.75 ± 11.47 a
Procyanidin B3	2.62 ± 1.53 a	2.99 ± 1.53 a	3.36 ± 2.21 a	4.52 ± 2.24 a
Epicatechin gallate	6.05 ± 5.55 a	3.31 ± 2.41 a	5.79 ± 6.58 a	6.10 ± 6.58 a
Flavonols				
Myricetin-3-glucoside	3.71 ± 2.4 a	3.97 ± 2.39 a	3.32 ± 1.66 a	2.67 ± 2.52 a
Myricetin-3-galactoside	3.31 ± 1.64 a	3.25 ± 2.14 a	3.55 ± 1.71 a	3.72 ± 2.76 a
Quercetin-3-glucoside	1.12 ± 0.37 a	1.42 ± 0.53 a	1.40 ± 0.37 a	1.21 ± 0.72 a
Quercetin	1.04 ± 0.93 a	0.32 ± 0.34 a	0.82 ± 0.86 a	0.72 ± 0.63 a

Table S5. Mean values of concentration (mg/L) and standard deviations (n=26) of the individual polyphenolic compounds belonging to different chemical families identified by HPLC-DAD in Pinot Noir red wines from different origin Valey

Phenolic compound	Bio-Bio (n=3)	Casablanca (n=8)	Limari (n=5)	San Antonio (n=3)	Valle central (n=7)
Glucosylated Anthocyanidins					
Delphinidin-3-glucoside	4,84 ± 2,35	5,5 ± 1,94	1,97 ± 0,35	7,43 ± 6,06	1,35 ± 0,24
Cyanidin-3-glucoside	6,32 ± 3,18	23,59 ± 4,88	19,36 ± 1,23	27,71 ± 13,93	15,11 ± 2,03
Petunidin-3-glucoside	4,45 ± 3,58	1,53 ± 0,65	1 ± 0,08	1,1 ± 0,4	0,96 ± 0,43
Peonidin-3-glucoside	6,79 ± 0,99	17,97 ± 3,71	20,21 ± 3,07	23,2 ± 10,76	7,39 ± 1,5
Malvidin-3-glucoside	82,63 ± 4,16	295,26 ± 47,73	278,49 ± 5,76	313,87 ± 122,84	180,07 ± 26,34
Benzoic acids					
Gallic acid	13,88 ± 1,87	13,57 ± 2,35	5,86 ± 0,8	8,12 ± 1,95	9,55 ± 1,26
Protocatechuic acid	0,94 ± 0,05	1,52 ± 0,13	1,32 ± 0,16	2,61 ± 0,52	1,51 ± 0,09
Hydroxicynnamic acids and derivatives					
Caffeic acid	12,49 ± 4,97	4,38 ± 0,37	1,64 ± 0,31	5,46 ± 2,17	2,87 ± 0,64
Caftaric acid	1,14 ± 0,61	1,88 ± 0,93	0,47 ± 0,04	2,73 ± 1,84	3,66 ± 1,93
Stilbenes					
Trans-resveratrol	0,66 ± 0,25	0,87 ± 0,09	1,05 ± 0,27	1,92 ± 0,91	*1,1 ± 0,07*
Flavan-3-ols					
(+)-Catechin	56,95 ± 24,26	53,42 ± 10,2	15,8 ± 4,21	15,45 ± 4,9	45,15 ± 11,62
(-)-Epicatechin	20,26 ± 10,61	18,26 ± 4,08	9,7 ± 2,5	8,06 ± 1,24	6,45 ± 1,26
Procyanidin B3	2,07 ± 1,18	4,34 ± 0,67	2,1 ± 0,46	2,5 ± 0,74	4,38 ± 0,81
Flavonols					
Myricetin-3-glucoside	1,81 ± 0,59	2,81 ± 0,62	4,06 ± 0,83	2,67 ± 1,87	4,48 ± 0,9
Myricetin-3-galactoside	1,73 ± 0,64	3,77 ± 0,75	4,06 ± 0,83	3,4 ± 1,69	3,5 ± 0,76
Quercetin-3-glucoside	0,26 ± 0,26	1,62 ± 0,2	1,43 ± 0,22	0,97 ± 0,23	1,02 ± 0,11
Quercetin	0,89 ± 0,7	0,65 ± 0,27	0,17 ± 0,01	0,78 ± 0,43	1,09 ± 0,23

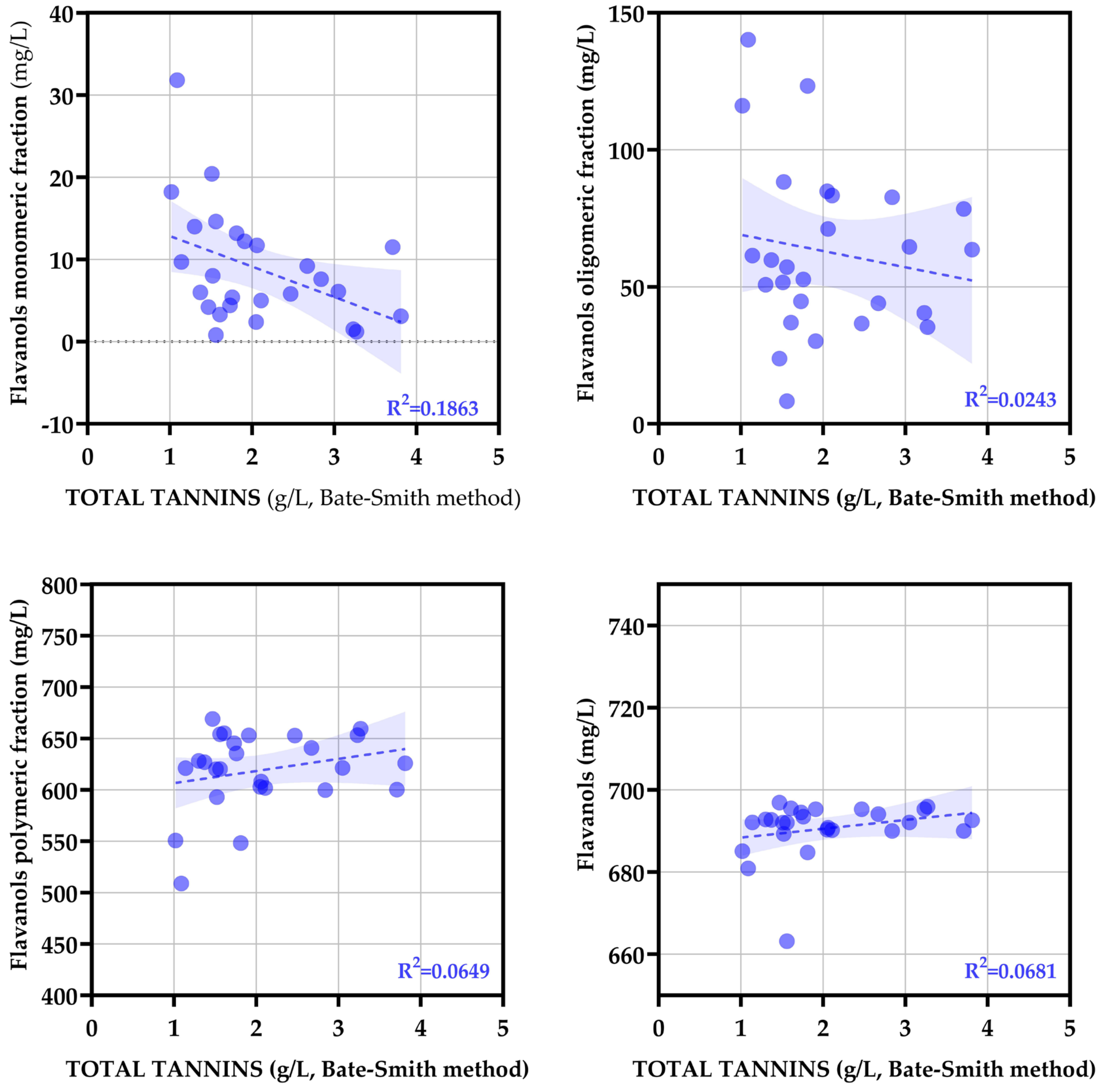

Figure S1. Relationship between total tannin concentration and the different proanthocyanidin (PA) fractions (monomeric, oligomeric, and polymeric) in commercial Chilean Pinot Noir wines. Scatter plots show the absence of a linear correlation between total tannins and individual PA fractions (r ≈ 0 in all cases). This lack of association suggests that total tannin content does not directly reflect the distribution of PA size classes, highlighting the presence of more complex and possibly independent mechanisms governing tannin polymerization, transformation, and refinement reactions during winemaking and wine aging.
